# On the Influence of Reward on Action-Effect Binding

**DOI:** 10.3389/fpsyg.2012.00450

**Published:** 2012-11-02

**Authors:** Paul S. Muhle-Karbe, Ruth M. Krebs

**Affiliations:** ^1^Department of Experimental Psychology, Ghent UniversityGhent, Belgium

**Keywords:** reward, motivation, ideomotor theory, action-effects, inter-individual differences

## Abstract

Ideomotor theory states that the formation of anticipatory representations about the perceptual consequences of an action [i.e., *action-effect (A-E) binding*] provides the functional basis of voluntary action control. A host of studies have demonstrated that A-E binding occurs fast and effortlessly, yet little is known about cognitive and affective factors that influence this learning process. In the present study, we sought to test whether the motivational value of an action modulates the acquisition of A-E associations. To this end, we linked specific actions with monetary incentives during the acquisition of novel A-E mappings. In a subsequent test phase, the degree of binding was assessed by presenting the former effect stimuli as task-irrelevant response primes in a forced-choice response task, absent reward. Binding, as indexed by response priming through the former action-effects, was only found for reward-related A-E mappings. Moreover, the degree to which reward associations modulated the binding strength was predicted by individuals’ trait sensitivity to reward. These observations indicate that the association of actions and their immediate outcomes depends on the motivational value of the action during learning, as well as on the motivational disposition of the individual. On a larger scale, these findings also highlight the link between ideomotor theories and reinforcement-learning theories, providing an interesting perspective for future research on anticipatory regulation of behavior.

## Introduction

The vast majority of actions we perform in everyday life are directed at producing a particular outcome in the environment. For instance, we may press a light switch because we want to illuminate the room, or boil water because we want to drink a cup of tea. In doing so, we effortlessly select actions that are appropriate for achieving a desired outcome. Accordingly, the ability to associate actions with their immediate and long-term consequences is a key mechanism for learning, and thus for flexible and adaptive control of behavior.

Ideomotor theory (IMT) constitutes the prevailing theoretical approach toward the role of effect anticipation in action control. The earliest versions of IMT can be traced back to the nineteenth century (Lotze, 1852; Harleß, 1861; James, 1890), and these ideas have undergone a renaissance in experimental psychology over the last decades (for recent reviews see Nattkemper et al., 2010; Shin et al., 2010; Pfister and Janczyk, 2012). In a nutshell, the core assumption of IMT is that actions and their perceptual outcomes are cognitively bound together. Performing an action (A) that produces a particular environmental effect (E) is assumed to lead to the formation of a common representation of the two events (“A-E binding”). Importantly, these bindings are conceived as bi-directional. Thus, internally anticipating a desired environmental effect directly activates the associated motor program, thereby promoting goal-directed behavior.

In the laboratory, this cardinal assumption of IMT is commonly assessed with so-called *induction paradigms* (Elsner and Hommel, 2001). Typically, participants first complete an acquisition phase to establish a novel association between simple actions and arbitrary sensory effects. For instance, participants may perform left- and right-hand button presses, each of which is contingently followed by a specific stimulus (e.g., left button → low-pitch tone, right button → high-pitch tone). In a subsequent test phase, the same responses are performed in a speeded forced-choice response task while the learned action-effects are presented as primes. Presupposing that participants have acquired bi-directional A-E bindings in the learning phase, the perception of a learned action-effect should directly activate the associated response, causing facilitation when the prime was previously the effect of the required response (compatible primes) and interference when the prime was previously the effect of a different response (incompatible primes). Over the last decade, this prediction has been confirmed in numerous studies employing a variety of response and effect modalities (e.g., Hommel, 1996; Elsner and Hommel, 2001; Beckers et al., 2002; Ziessler and Nattkemper, 2002; Kunde, 2004; Ziessler et al., 2004; Herwig et al., 2007).

Interestingly, once A-E knowledge has been acquired, the priming of a response via the activation of an associated perceptual representation seems to occur highly automatically, without requiring further cognitive mediation. For instance, it also occurs in conditions in which effect primes are entirely task-irrelevant (Hommel, 1996) and even when the primes are presented subliminally so that they cannot be consciously perceived (Kunde, 2004). On the other hand, relatively little is known about the factors that contribute to the *acquisition* of this kind of knowledge. Elsner and Hommel (2004) have investigated situational determinants of A-E binding, demonstrating that it critically depends on the temporal contiguity and the probabilistic contingency between actions and their effects. In other words, A-E binding diminishes with increasing delays between the two events, as well as with reduced predictability of a unique effect. Other studies have shown that cognitive factors such as the internal selection of an action may influence the strength of A-E binding during the acquisition phase (Ziessler et al., 2004; Herwig et al., 2007; Herwig and Waszak, 2009; Kühn et al., 2009; but see Pfister et al., 2011).

Here, we wanted to examine whether the acquisition of A-E bindings can moreover be modulated by factors related to the *motivational value* of an action. It is well established that monetary incentives can be used to modulate a wide range of human cognitive functions including visual discrimination, conflict resolution, and long-term memory encoding (Wittmann et al., 2005; Engelmann and Pessoa, 2007; Padmala and Pessoa, 2008; Krebs et al., 2012). In these paradigms, reward is typically associated with specific trial types, stimulus types, or entire task blocks, in such a way that the participant is rewarded for correct and/or fast executions of the required response. As such, these stimulus-reward associations are in most cases compatible with the task goal, which generally results in a facilitation of response execution. However, we recently showed that reward associations can also have detrimental effects upon response execution if they trigger specific response tendencies that are incompatible with the task goal (Krebs et al., 2010, 2011). Another line of research has demonstrated that not only perceptual but also affective features of outcomes are bound to the actions that produce them. Specifically, in a study by Beckers et al. (2002), one of two responses in a free-choice task was always associated with an electrocutaneous stimulation (negative valence), while the other was not (positive valence). In the subsequent test phase, responses to target words were facilitated if their semantic valence was compatible with the effect previously associated with this response (Beckers et al., 2002). Similar effects of “affective compatibility” have been observed in a recent study by Eder et al. (2012). The authors showed that preparing a response to a picture of positive or negative valence interfered with the actual execution of a subsequent response to a word of similar valence. This suggests that action planning involves the activation of associated affective features, making them less accessible to other responses that share this feature.

While these findings highlight that affective codes are a part of the mental representation of an action, we wanted to further investigate whether motivational values of an action would modulate the degree of A-E binding – a notion which has not yet been tested. To this end, we associated two out of four actions with monetary incentives during the acquisition phase of an induction paradigm. In the subsequent test phase, we assessed the influence of compatible and incompatible effect primes, which could be related to former reward or to no reward, in the absence of any further monetary reinforcement. Considering previous evidence that affective feedback stimuli can strengthen sensorimotor integration (Colzato et al., 2007a; Waszak and Pholulamdeth, 2009), and that reward-related stimuli can prime response tendencies even if they are task-irrelevant (Krebs et al., 2010), we predicted that binding would be stronger for rewarded A-E mappings as compared to unrewarded mappings. This should be reflected in increased compatibility effects for primes that were previously related to a rewarded action, and would provide direct evidence that the acquisition of action-effect knowledge can be modulated by changes in the motivational value of an action and its consequence.

## Materials and Methods

### Participants and procedure

Twenty-six undergraduate students from Ghent University (eight male, four left-handed) participated in the study (mean age = 18.72 years; SD = 1.02). They all had normal or corrected to normal vision, gave written and informed consent to participate, and were naive to the rationale of the experiment. Stimuli were presented on a PC with a 17” monitor and responses were given with both index and middle fingers using the buttons “A,” “S,” “K,” and “L” on a QWERTY computer keyboard. Following the experiment, participants completed the Behavioral-Inhibition and Behavioral-Activation Scales (BIS/BAS; Carver and White, 1994) to assess individual sensitivity to reward. The whole procedure lasted approximately 30 min. All participants received a basic compensation of 4 euro and an average performance-related bonus of 2.5 euro.

### Experimental design

In line with previous research on A-E binding, the experiment consisted of two phases. First, participants completed an acquisition phase to establish learning of novel A-E mappings. For the given purpose, we manipulated the reward value of these mappings by associating half of them with monetary incentives. In the subsequent test phase, in which participants could no longer earn bonuses, the degree of A-E binding was assessed by presenting the previous action-effects as task-irrelevant response primes. Based on our assumption that reward would modulate the binding between actions and their effects during the acquisition phase, we predicted that reward-related primes would induce greater incompatibility effects as compared to reward-unrelated primes in the test phase.

### Acquisition phase

**Figure 1:**
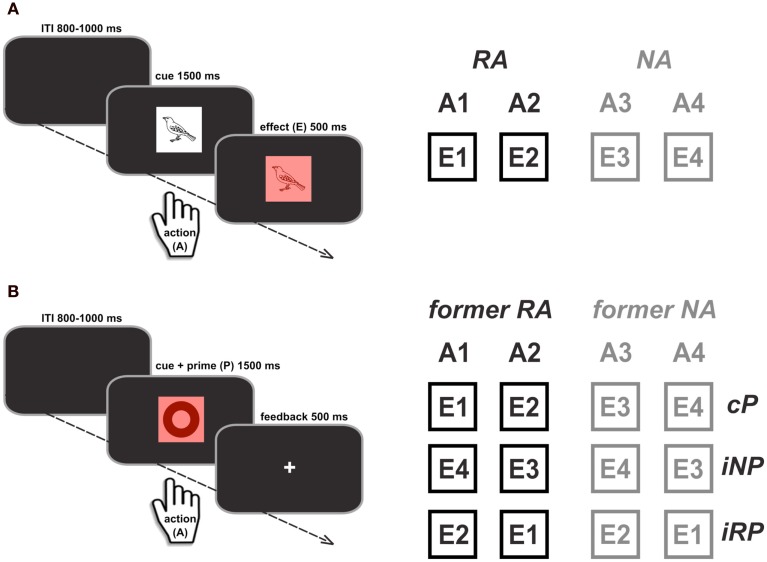
**Illustration of the experimental paradigm in the acquisition phase (A) and test phase (B)**. During acquisition, two out of the four actions were associated with reward (RA vs. NA). The unique effects (E1–E4) that were produced by specific actions (A1–A4) were used as response primes in the subsequent test phase. Primes could be either compatible with the required response (cP) or incompatible (shown for one exemplary A-E mapping). Due to the reward manipulation during acquisition, incompatible primes in the test phase could be either related to reward (iRP) or to no-reward (iNP) effects. The primes, however, were entirely irrelevant to the task and no longer predictive of reward in the test phase.

The acquisition phase consisted of a forced-choice reaction time (RT) task with four different responses. Within a given block, each response was consistently mapped onto one specific picture (response cue) taken from a set of line drawings (Snodgrass and Vanderwart, 1980). At the beginning of each block, the four specific response cues were presented on the screen along with their associated responses. In each trial, after a variable intertrial interval (ITI) of 800–1000 ms, one of the cues was centrally presented for the maximum duration of 1500 ms (Figure 1A, left panel). Immediately after a response was given, or the maximum duration was reached, a colored square was displayed for 500 ms in the background of the cue, serving as a visual action-effect (see Wolfensteller and Ruge, 2011 for a similar procedure). In case of correct responses, the background color was response-specific (red, green, blue, or yellow), and in case of incorrect or late responses (>1500 ms) the background square turned gray. Participants were instructed to respond to the cues as quickly and as accurately as possible. Furthermore, they were told that the background color would indicate if their response on a given trial was correct and within the critical time window. Importantly, the picture category of the current cue (living animals vs. non-living objects) indicated whether a correct response (action, A) would be rewarded (reward action, RA) or not (no-reward action, NA). For each correct response that was given within the maximum time window of 1500 ms, 10 points were automatically added to the participants’ score, which determined the total gain in Euro cents (0.5 euro per 200 points). The cue-category association with reward was counterbalanced across participants and cue categories were equally assigned to both hands and to index and middle fingers. In each block, a novel set of cue pictures was introduced in order to keep the task at a constant level of difficulty. However, mappings between cue categories and responses, and between responses and effect colors were constant for each participant (counterbalanced across participants). Overall, participants worked through four blocks of 60 trials, resulting in 120 reward trials and 120 no-reward trials performed with two fingers each.

### Test phase

In the test phase, participants completed a similar RT task using the same responses as before. They were told that there was no longer anything to win, but that they should continue to respond as quickly and accurately as possible. Importantly, responses were cued by a new set of pictures that were not associated with the previous cue categories (abstract symbols from the creative symbol collection of Matton images^1^). The new cue-category was introduced to eliminate a potentially confounding influence of stimulus-effect associations on task performance in the test phase (cf. Wolfensteller and Ruge, 2011). To probe the degree of A-E binding, the previous action-effects were now presented as response primes (i.e., displayed as squares in the background at 100 ms prior to cue onset until the offset of the cue). Participants were instructed that the colors were irrelevant for the task at hand and should thus be ignored. Analogous to the acquisition phase, cues remained on the screen for a maximum duration of 1500 ms. After a response was given or the maximum duration was reached, performance feedback was presented centrally for 500 ms, with a “+” indicating correct and fast responses and a “−” indicating response errors or omissions (Figure 1B). All possible combinations of response cues and primes were presented equally often, resulting in three types of primes: (1) compatible primes (cP, compatible to previous A-E mapping), (2) incompatible reward-related primes (iRP, effect of a different previously reward-related response), and (3) incompatible no-reward primes (iNP, effect of a different previously reward-unrelated response). Moreover, responses themselves could be distinguished based on whether they had been related to reward in the acquisition phase (former RA) or not (former NA). Altogether, participants completed eight trials of each prime response combination, resulting in a total of 128 trials (32 cP, 48 iRP, 48 iNP).

## Results

### Acquisition phase

As expected, participants’ responses were faster on trials with RA than on trials with NA (RA < NA; *t* = 6.58, *p* < 0.001; Table 1), confirming that cue-reward associations facilitated performance in the respective trials. Overall, participants responded highly accurately with a small numerical but non-significant difference between reward and no-reward trials (96.8 vs. 95.4%; *p* > 0.1).

**Table 1:** **Behavioral performance in acquisition and test phase**.

	RT ms (SE)		Accuracy% (SE)	
**Acquisition**	**RA**	**NA**	**RA**	**NA**
	553.1 (10.0)	608.2 (13.9)	96.8 (0.6)	95.4 (0.7)
**Test**	**Former RA**	**Former NA**	**Former RA**	**Former NA**
cP	580.6 (14.6)	573.2 (10.6)	96.5 (1.0)	98.3 (0.6)
iNP	575.7 (11.0)	580.0 (10.9)	97.2 (0.6)	96.8 (1.0)
iRP	574.0 (10.9)	593.5 (12.7)	95.6 (1.2)	97.5 (0.6)

*RA, reward action; NA, no-reward action; cP, compatible prime; iNP, incompatible no-reward prime; iRP, incompatible reward prime; SE, standard error of the mean*.

### Test phase response times (RTs)

**Figure 2:**
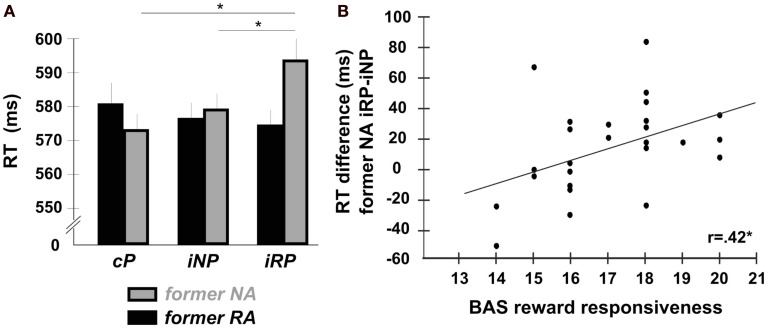
**Influence of reward-related primes in the test phase**. **(A)** Despite being entirely irrelevant to the task and being no longer predictive of reward, incompatible reward-related primes (iRP) differentially increased RTs to new cues in the test phase. This effect was unique to *former* NA responses, in which the required action was never associated with actual reward. Error bars depict the standard error of the mean (SE) for within-subject comparisons. **(B)** The size of the RT-differences on trials with incompatible reward-related primes compared to trials with incompatible reward-unrelated primes (iRP > INP) on *former* NA trials correlated with participants dispositional responsiveness to reward.

Mean RTs of correct responses in the test phase were analyzed using a 2 × 3 repeated-measures analysis of variance (rANOVA) with reward-relatedness of the action (RA vs. NA) and prime compatibility (cP vs. iNP vs. iRP) as within-subject factors (Figure 2A; Table 1). The assumption of sphericity for the rANOVAs was tested using Mauchley’s method. Since no significant violations were observed (all *W*-values > 0.8, *p* > 0.2), uncorrected *F* statistics are reported in the results. There was neither a main effect of reward-relatedness [*F*(1,25) = 0.44, *p* = 0.512, $\eta_{p}^{2}=$ 0.017] nor a main effect of prime compatibility [*F*(2,50) = 1.24, *p* = 0.298, $\eta_{p}^{2}=$ 0.017] alone, but a significant interaction of the two factors [*F*(2,50) = 3.58, *p* = 0.035, $\eta_{p}^{2}=$ 0.125]. Post hoc *t*-tests employed to test the nature of this interaction revealed that for former NA, RTs were significantly slower when primed with incompatible reward-related effects compared to incompatible reward-unrelated effects [iRP > iNP; *t*(25) = 2.18, *p* = 0.031, Cohen’s *d* = 0.225], as well as compared to compatible primes [iRP > cP; *t*(25) = 3.12, *p* = 0.005, *d* = 0.341]. The difference between iNP and cP was not significant [*t*(25) = 0.966, *p* = 0.344; *d* = 0.122]. By contrast, for former RA, RTs did not differ at all across prime conditions (all *p*-values > 0.4).

### Test phase accuracy

An identical rANOVA on the response accuracy revealed no main effects of reward-relatedness of the action or prime compatibility, and no interaction of the two factors (all *p*-values > 0.1). This indicates that the conditions did not differ with regard to the *absolute* percentages of errors. We conducted an additional analysis of the *relative* percentages (i.e., ratios) of different error types across conditions to explore whether the ratio of prime-consistent errors would be increased in iRP-trials. This would support the notion that the perception of former reward-related effects indeed induced a specific, albeit false, action in the test phase (see Schmidt and De Houwer, 2011 for a similar analysis of different error types). To this end, we distinguished between *prime-consistent errors*, defined as erroneous responses that were consistent with the incompatible prime on a given trial, and *prime-inconsistent errors*, defined as erroneous responses that were not consistent with the incompatible prime, i.e., random errors. Observed ratios for prime-consistent errors were compared with a baseline of 33.3% that would be expected under a random error distribution with only one out of three possible false responses being prime-consistent. It should be noted that this analysis is limited in two ways, and must hence be considered exploratory: first, due to the nature of the paradigm, only incompatible conditions could be included, as no prime-consistent errors could be made on compatible trials. Second, the analysis could only be performed on a subset of participants, i.e., those who committed errors in the respective conditions (former RA trials: *N* = 13; former NA trials: *N* = 11). Ratios of prime-consistent errors were significantly increased in only one condition, namely on trials in which former no-reward responses were primed with incompatible reward-related effects [iRP: 62 vs. 33.3%, *t*(10) = 2.3, *p* = 0.042].

### Individual reward responsiveness

Our final analysis was concerned with the relation of participants’ task performance to inter-individual differences in reward responsiveness. If the observed priming effect indeed reflects reward-driven strengthening of A-E bindings, then the size of this effect may be related to participants’ dispositional sensitivity to rewarding events. To this end, we correlated individual RT-differences between iRP-trials and iNP-trials with the individual scores on the reward responsiveness subscale of the BIS/BAS (Carver and White, 1994), which is thought to reflect an individual’s dispositional responsiveness to rewarding events. In the present sample, individual reward responsiveness scores varied between 14 and 20 (mean score = 17, SD = 1.74). We observed a significant correlation between RT difference values (NA-iRP minus NA-iNP) and the reward responsiveness subscale across all 26 participants [r(24) = 0.42, *p* = 0.030, two-tailed], indicating that those participants who reported being more responsive to reward in general showed a greater slowing on NA-iRP-trials compared to NA-iNP-trials (Figure 2B).

## Discussion

The present study investigated the influence of reward on A-E binding. We hypothesized that the intrinsic tendency to associate actions with their contingent outcomes could be influenced by assigning motivational values to specific actions. Following an acquisition phase in which half of the applied A-E mappings were related to monetary incentives, the strength of A-E binding was assessed in a test phase by presenting the former action-effects as task-irrelevant primes.

Altogether, three major findings were evident, all of which confirmed our prediction. First, and most importantly, induction effects were only found for primes that had been associated with reward during acquisition, providing direct evidence that reward strengthens the association between actions and their outcomes. Note that these differential effects occurred although the primes were entirely irrelevant to the task at hand and they were no longer predictive of any reward, which highlights the automatic nature of the binding process. Second, besides slower RTs on trials with correct responses, the same incompatible reward-related primes also increased the ratio of prime-consistent errors compared to a random distribution. This finding nicely illustrates the specificity of the interference effect at the response level and thus directly mirrors the concept of bi-directional action-effect representations in the framework of IMT. Third, inter-individual differences in reward responsiveness predicted the size of differential binding effects for reward-related and reward-unrelated primes. This finding further supports the idea that the observed induction effect with reward-related primes is related to incentive value representations of specific A-E bindings, which likely vary across individuals. Such a pattern is highly consistent with previously reported correlations between reward-sensitivity traits and actual behavioral responsiveness to reward (Kambouropoulos and Staiger, 2004), as well as between reward-related performance facilitation and neural activity in brain regions implicated in reward processing (Locke and Braver, 2008).

It is, however, important to consider to what extent the observed induction effect with reward-related primes indeed reflects a modulation of A-E binding in the acquisition phase. It could be argued that the influence of former reward effects arises from prioritized processing of a salient stimulus. Several possible outcomes are possible: for instance, stimulus processing could be generally facilitated by the salient effect, similar to effects of reward-related colors in a visual search array (Kiss et al., 2009). Such facilitation should, however, result in faster rather than slower response execution due to the advanced access to stimulus information. The salient effect color could also lead to a general distraction form the main task. Such effects have been demonstrated by using salient stimuli as irrelevant flankers in a target-discrimination task (Serences et al., 2005), as well as for reward-related colors that were presented at irrelevant positions in a visual search task (Hickey et al., 2010). Finally, participants could have experienced some kind of frustration in trials displaying former reward-related effects in the test phase, as they could no longer earn bonus money. In turn, frustration could cause unspecific attentional distraction. Importantly, however, all these forms of attentional distraction are unlikely to trigger specific erroneous response tendencies, which is suggested by the result of the exploratory error types analysis in the present study.

It is moreover key to exclude the possibility that the observed differential effect in the test phase is an artefact of the individuals’ performance during the acquisition phase. As noted above, there was no difference in performance accuracy between reward-related and unrelated trials. Thus, participants experienced a similar number of A-E couplings in both conditions. Furthermore, participants responded faster in reward-related trials in the acquisition phase. This nicely illustrates that participants were indeed motivated by the prospect of reward and optimized their performance accordingly (Krebs et al., 2010; Schmidt et al., 2012). It could thus be argued that the observed binding for reward-related A-E mappings is a mere consequence of participants allocating more attention to the reward-related color effects during acquisition. Although recent evidence indicates that directing the focus of attention toward action outcomes during the acquisition phase does not automatically facilitate A-E binding (Herwig and Waszak, 2009), future research should certainly specify the mechanisms by which reward modulates A-E binding and to what extent it relies on the modulation of attentional mechanisms.

An additional interesting observation was that responses that had been associated with reward during acquisition were unaffected by prime compatibility in the test phase. Considering that reward-predictive stimuli have not only been shown to increase attention but also to strengthen the associated response pathways (e.g., Krebs et al., 2011; Schmidt et al., 2012), it is feasible to assume that former reward-associated responses in the current study are less prone to interfering information, namely incompatible effect primes.

Another noteworthy finding in the present study was the absence of significant compatibility effects with reward-unrelated primes. This non-finding is rather surprising since binding for unrewarded effects has already been demonstrated frequently in the literature (e.g., Hommel et al., 2003 or Hoffmann et al., 2009). However, the absence of compatibility effects for reward-unrelated primes may be associated with methodological aspects of the present experimental design. First, our study employed visual action-effects, which have been shown to be less salient than auditory action-effects, thereby leading to weaker A-E binding (Kunde, 2001; Dutzi and Hommel, 2009). Moreover, the paradigm was designed to minimize the influence of possibly confounding factors that could artificially inflate the size of induction effects. For instance, we excluded an influence of cue-effect associations by introducing a novel set of pictures as cues in the test phase. Furthermore, the present study employed a full combination of primes and responses, i.e., each effect occurred multiple times both as compatible and as incompatible prime. By using this design, the influence of each particular effect is necessarily weakened in comparison with classical paradigms that present effect stimuli as either only compatible or only incompatible primes in the test phase (cf. Elsner and Hommel, 2004; Wolfensteller and Ruge, 2011). A final paradigmatic aspect relates to the timing of prime presentation relative to the onset of the response cues. Recently, Ziessler and Nattkemper (2011) employed a systematic manipulation of the stimulus-onset asynchrony (SOA) between effect primes and response cues. Effects of prime compatibility were only observed when the primes were presented *after* cue onset. Thus, the absence of priming effects for reward-unrelated effects in the present study could be partly due to the fact that the primes may not have been presented at the time of their maximal effectiveness.

From a more general perspective, it is moreover a common observation that the introduction of reward signals not only modulates performance in those trials that are subject to actual reward, but it also modifies the general task context, resulting in altered performance on the no-reward trials, as compared to a “neutral” task-contexts without reward (e.g., Braem et al., 2012). Thus, in the present study, the presence of reward in the acquisition phase may have influenced participants’ experience of the unrewarded A-E mappings as well. It could be argued that unrewarded effects in a reward context may be perceived as less significant. Specifically, it has been demonstrated that behavioral and neural influences of high-reward vs. low-reward stimuli critically depend on the overall context, i.e., the differences between trial types become more distinct in a general reward context (Delgado et al., 2004). Such a relative “devaluation” of unrewarded effects may counteract A-E binding in the present paradigm, such that for an action which does not produce an explicitly positive outcome, a bi-directional binding of the two events might be attenuated. Future research could explore this question by explicitly introducing reward as well and punishment signals during the acquisition of A-E associations.

Future research should also specify the precise mechanisms by which reward enhances the association strength of motor representations and representations of the respective sensory outcomes. It is known from numerous studies employing reward-modulated paradigms that reward associations can influence cognitive functions and behavior via diverse mechanisms (Pessoa, 2009; Pessoa and Engelmann, 2010). Among them are the prioritization of perceptual processing and the enforcement of specific response tendencies, as well as the increase of cognitive and physical effort to perform the task and the change of long-term stimulus representations. While conclusive statements about the underlying mechanism may not be warranted based on the present data, it appears likely that reward modulates the behavioral relevance of both an action and its consequence, which may in turn enforce the joint coding of the two events.

With regard to the neural level, dopamine has been proposed to underlie the formation of sensorimotor associations (Colzato et al., 2007a). Considering that reward-predicting stimuli are known to trigger dopaminergic activity (Knutson and Gibbs, 2007; Schott et al., 2008), it is likely that the reward-related effect in our own study is mediated by dopamine as well. Future studies will be needed to illuminate this relationship further, e.g., by assessing markers of individual dopamine levels, such as the spontaneous eye-blink rate, as covariates (Colzato et al., 2007b; Aarts et al., 2012), or by employing a similar paradigm in individuals with specific genotypes or clinical conditions promoting differential striatal dopamine levels (Schott et al., 2007; Yacubian et al., 2007).

## Conflict of Interest Statement

The authors declare that the research was conducted in the absence of any commercial or financial relationships that could be construed as a potential conflict of interest.

## Footnotes

^1^http://www.mattonimages.de/bilder/cd/ingram_publishing/creative_symbol_collection

## References

[B1] Aarts H. Bijleveld E. Custers R. Dogge M. Deelder M. Schutter D. (2012). Positive priming and intentional binding: eye-blink rate predicts reward information effects on the sense of agency. Soc. Neurosci. 7, 105–112 10.1080/17470919.2011.590602 21936738

[B2] Beckers T. De Houwer J. Eelen P. (2002). Automatic integration of non-perceptual action effect features: the case of the associative affective Simon effect. Psychol. Res. 66, 166–173 10.1007/s00426-002-0090-9 12192445

[B3] Braem S. Verguts T. Roggeman C. Notebaert W. (2012). Reward modulates adaptations to conflict. Cognition 125, 324–332 10.1016/j.cognition.2012.07.015 22892279

[B4] Carver C. S. White T. L. (1994). Behavioral inhibition, behavioral activation, and affective responses to impending reward and punishment: the BIS/BAS Scales. J. Pers. Soc. Psychol. 67, 319–333 10.1037/0022-3514.67.2.319

[B5] Colzato L. S. van Wouwe N. C. Hommel B. (2007a). Feature binding and affect: emotional modulation of visuo-motor integration. Neuropsychologia 45, 440–446 10.1016/j.neuropsychologia.2007.03.004 16926036

[B6] Colzato L. S. van Wouwe N. C. Hommel B. (2007b). Spontaneous eyeblink rate predicts the strength of visuomotor binding. Neuropsychologia 45, 2387–2392 10.1016/j.neuropsychologia.2007.03.004 17433381

[B7] Delgado M. R. Stenger V. A. Fiez J. A. (2004). Motivation-dependent responses in the human caudate nucleus. Cereb. Cortex 14, 1022–1030 10.1093/cercor/bhh062 15115748

[B8] Dutzi I. B. Hommel B. (2009). The microgenesis of action-effect binding. Psychol. Res. 73, 425–435 10.1007/s00426-008-0161-7 18810487

[B9] Eder A. B. Musseler J. Hommel B. (2012). The structure of affective action representations: temporal binding of affective response codes. Psychol. Res. 76, 111–118 10.1007/s00426-011-0327-6 21442406

[B10] Elsner B. Hommel B. (2001). Effect anticipation and action control. J. Exp. Psychol. Hum. Percept. Perform. 27, 229–240 10.1037/0096-1523.27.1.229 11248937

[B11] Elsner B. Hommel B. (2004). Contiguity and contingency in action-effect learning. Psychol. Res. 68, 138–154 10.1007/s00426-003-0151-8 14685854

[B12] Engelmann J. B. Pessoa L. (2007). Motivation sharpens exogenous spatial attention. Emotion 7, 668–674 10.1037/1528-3542.7.4.875 17683222

[B13] Harleß E. (1861). Der Apparat des Willens. Zeitschrift für Philosophie und Philosophische Kritik 38, 50–73

[B14] Herwig A. Prinz W. Waszak F. (2007). Two modes of sensorimotor integration in intention-based and stimulus-based actions. Q. J. Exp. Psychol. 60, 1540–1554 10.1080/17470210601119134 17853217

[B15] Herwig A. Waszak F. (2009). Intention and attention in ideomotor learning. Q. J. Exp. Psychol. 62, 219–227 10.1080/17470210802373290 18932052

[B16] Hickey C. Chelazzi L. Theeuwes J. (2010). Reward changes salience in human vision via the anterior cingulate. J. Neurosci. 30, 11096–11103 10.1523/JNEUROSCI.1026-10.2010 20720117 PMC6633486

[B17] Hoffmann J. Lenhard A. Sebald A. Pfister R. (2009). Movements or targets: what makes an action in action-effect learning? Q. J. Exp. Psychol. 62, 2433–2449 10.1080/17470210902922079 19526438

[B18] Hommel B. (1996). The cognitive representation of action: automatic integration of perceived action effects. Psychol. Res. 59, 176–186 10.1007/BF00425832 8923816

[B19] Hommel B. Alonso D. Fuentes L. (2003). Acquisition and generalization of action effects. Vis. cogn. 10, 965–986 10.1080/13506280344000176

[B20] James W. (1890). The principles of psychology. New York: Holt

[B21] Kambouropoulos N. Staiger P. K. (2004). Reactivity to alcohol-related cues: relationship among cue type, motivational processes, and personality. Psychol. Addict. Behav. 18, 275–283 10.1037/0893-164X.18.3.275 15482083

[B22] Kiss M. Driver J. Eimer M. (2009). Reward priority of visual target singletons modulates event-related potential signatures of attentional selection. Psychol. Sci. 20, 245–251 10.1111/j.1467-9280.2009.02281.x 19175756 PMC2645377

[B23] Knutson B. Gibbs S. E. (2007). Linking nucleus accumbens dopamine and blood oxygenation. Psychopharmacology (Berl.) 191, 813–822 10.1007/s00213-006-0686-7 17279377

[B24] Krebs R. M. Boehler C. N. Egner T. Woldorff M. G. (2011). The neural underpinnings of how reward associations can both guide and misguide attention. J. Neurosci. 31, 9752–9759 10.1523/JNEUROSCI.0732-11.2011 21715640 PMC3142621

[B25] Krebs R. M. Boehler C. N. Roberts K. C. Song A. W. Woldorff M. G. (2012). The involvement of the dopaminergic midbrain and cortico-striatal-thalamic circuits in the integration of reward prospect and attentional task demands. Cereb. Cortex 22, 607–615 10.1093/cercor/bhr134 21680848 PMC3278318

[B26] Krebs R. M. Boehler C. N. Woldorff M. G. (2010). The influence of reward associations on conflict processing in the Stroop task. Cognition 117, 341–347 10.1016/j.cognition.2010.08.018 20864094 PMC2967668

[B27] Kühn S. Elsner B. Prinz W. Brass M. (2009). Busy doing nothing: evidence for nonaction – effect binding. Psychon. Bull. Rev. 16, 542–549 10.3758/PBR.16.3.542 19451382

[B28] Kunde W. (2001). Response-effect compatibility in manual choice reaction tasks. J. Exp. Psychol. Hum. Percept. Perform. 27, 387–394 10.1037/0096-1523.27.2.387 11318054

[B29] Kunde W. (2004). Response priming by supraliminal and subliminal action effects. Psychol. Res. 68, 91–96 10.1007/s00426-003-0156-3 14634809

[B30] Locke H. S. Braver T. S. (2008). Motivational influences on cognitive control: behavior, brain activation, and individual differences. Cogn. Affect. Behav. Neurosci. 8, 99–112 10.3758/CABN.8.1.99 18405050

[B31] Lotze R. H. (1852). Medizinische Psychologie oder die Physiologie der Seele. Leipzig: Weidmann’sche Buchhandlung

[B32] Nattkemper D. Ziessler M. Frensch P. A. (2010). Binding in voluntary action control. Neurosci. Biobehav. Rev. 34, 1092–1101 10.1016/j.neubiorev.2009.12.013 20036685

[B33] Padmala S. Pessoa L. (2008). Affective learning enhances visual detection and responses in primary visual cortex. J. Neurosci. 28, 6202–6210 10.1523/JNEUROSCI.1233-08.2008 18550762 PMC2673575

[B34] Pessoa L. (2009). How do emotion and motivation direct executive control? Trends Cogn. Sci. (Regul. Ed.) 13, 160–166 10.1016/j.tics.2009.01.006 19285913 PMC2773442

[B35] Pessoa L. Engelmann J. B. (2010). Embedding reward signals into perception and cognition. Front. Neurosci. 4:17 10.3389/fnins.2010.00017 20859524 PMC2940450

[B36] Pfister R. Janczyk M. (2012). Harless’ apparatus of will: 150 years later. Psychol. Res. 76, 561–565 10.1007/s00426-011-0362-3 21748464 PMC3419348

[B37] Pfister R. Kiesel A. Hoffmann J. (2011). Learning at any rate: action-effect learning for stimulus-based actions. Psychol. Res. 75, 61–65 10.1007/s00426-010-0288-1 20490862

[B38] Schmidt J. De Houwer J. (2011). Now you see it, now you don’t: Controlling for contingencies and stimulus repetitions eliminates the Gratton effect. Acta Psychol. (Amst) 138, 176–186 10.1016/j.actpsy.2011.06.002 21745649

[B39] Schmidt L. Lebreton M. Clery-Melin M. L. Daunizeau J. Pessiglione M. (2012). Neural mechanisms underlying motivation of mental versus physical effort. PLoS Biol. 10, e1001266 10.1371/journal.pbio.1001266 22363208 PMC3283550

[B40] Schott B. H. Minuzzi L. Krebs R. M. Elmenhorst D. Lang M. Winz O. H. (2008). Mesolimbic functional magnetic resonance imaging activations during reward anticipation correlate with reward-related ventral striatal dopamine release. J. Neurosci. 28, 14311–14319 10.1523/JNEUROSCI.2058-08.2008 19109512 PMC6671462

[B41] Schott B. H. Niehaus L. Wittmann B. C. Schutze H. Seidenbecher C. I. Heinze H. J. (2007). Ageing and early stage Parkinson’s disease affect separable neural mechanisms of mesolimbic reward processing. Brain 130, 2412–2424 10.1093/brain/awl369 17626038

[B42] Serences J. T. Shomstein S. Leber A. B. Golay X. Egeth H. E. Yantis S. (2005). Coordination of voluntary and stimulus-driven attentional control in human cortex. Psychol. Sci. 16, 114–122 10.1111/j.0956-7976.2005.00791.x 15686577

[B43] Shin Y. K. Proctor R. W. Capaldi E. J. (2010). A review of contemporary ideomotor theory. Psychol. Bull. 136, 943–974 10.1037/a0021628 20822210

[B44] Snodgrass J. G. Vanderwart M. (1980). A standardized set of 260 pictures: norms for name agreement, image agreement, familiarity, and visual complexity. J. Exp. Psychol. Hum. Learn. 6, 174–215 10.1037/0278-7393.6.2.174 7373248

[B45] Waszak F. Pholulamdeth V. (2009). Episodic S-R bindings and emotion: about the influence of positive and negative action effects on stimulus-response associations. Exp. Brain Res. 194, 489–494 10.1007/s00221-009-1745-1 19266189

[B46] Wittmann B. C. Schott B. H. Guderian S. Frey J. U. Heinze H. J. Duzel E. (2005). Reward-related FMRI activation of dopaminergic midbrain is associated with enhanced hippocampus-dependent long-term memory formation. Neuron 45, 459–467 10.1016/j.neuron.2005.01.010 15694331

[B47] Wolfensteller U. Ruge H. (2011). On the timescale of stimulus-based action-effect learning. Q. J. Exp. Psychol. 64, 1273–1289 10.1080/17470218.2010.546417 21416458

[B48] Yacubian J. Sommer T. Schroeder K. Glascher J. Kalisch R. Leuenberger B. (2007). Gene-gene interaction associated with neural reward sensitivity. Proc. Natl. Acad. Sci. U.S.A. 104, 8125–8130 10.1073/pnas.0702029104 17483451 PMC1864910

[B49] Ziessler M. Nattkemper D. (2002). “Effect Anticipation in Action Planning: Anticipative Learning of Action Effects,” in Common Mechanisms in Perception and Action: Attention and Performance, Vol. XIX, eds Prinz W. Hommel B. (Oxford University Press, Oxford), 645–672

[B50] Ziessler M. Nattkemper D. (2011). The temporal dynamics of effect anticipation in course of action planning. Q. J. Exp. Psychol. 64, 1305–1326 10.1080/17470218.2011.553067 21416456

[B51] Ziessler M. Nattkemper D. Frensch P. A. (2004). The role of anticipation and intention in the learning of effects of self-performed actions. Psychol. Res. 68, 163–175 10.1007/s00426-003-0153-6 14634810

